# Effects of Freeze–Thaw Cycles on Axial Compression Behaviors of UHPC-RC Composite Columns

**DOI:** 10.3390/ma17081843

**Published:** 2024-04-17

**Authors:** Shuling Gao, Leyu Liu

**Affiliations:** 1 School of Civil and Transportation Engineering, Hebei University of Technology, Tianjin 300401, China; lly_llzxc@163.com; 2 Civil Engineering Technology Research Center of Hebei Province, Tianjin 300401, China

**Keywords:** UHPC-RC composite column, axial compression properties, high-strength stirrups, stirrup spacing

## Abstract

Ultra-high performance concrete (UHPC) with excellent durability has broad application prospects in improving the durability of reinforced concrete (RC) structures. To clarify the influence of freeze–thaw cycles on the axial compression performance of UHPC-RC composite columns, axial compression tests were carried out on composite columns with different cycles (0, 100, 200, 300 cycles) and stirrup spacing (35, 70, 105 mm). The results showed that the UHPC shell did not fall off when the composite column was destroyed, even in the freeze–thaw environment. Under the action of freeze–thaw cycles, the peak load $N_{u,t}$ and initial elastic modulus E of the composite column decreased, but the ductility coefficient μ increased. Increasing the stirrup spacing could significantly improve the ductility of the composite column. After 100 freeze–thaw cycles, the ductility coefficient μ of the 35 mm stirrup spacing specimen was 112.6% higher than that of the 105 mm specimen. A prediction model for the bearing capacity of UHPC-RC composite columns under freeze–thaw cycles was established, and the predicted results were in good agreement with the experimental results. This study lays a theoretical and experimental foundation for the application and design of UHPC-RC composite columns in the freeze–thaw environment.

## 1. Introduction

The wading structures serving in cold regions, such as bridge piers and wharf support columns, may encounter freeze–thaw disasters during service. The freeze–thaw cycle will cause a decrease in the mechanical properties of concrete and the bonding performance between steel and concrete, which seriously affects the service life of the structure, and the maintenance cost is high every year [1]. Therefore, it is becoming increasingly urgent to solve the problem of structural performance degradation caused by freeze–thaw cycles.

To solve the problems caused by freeze–thaw damage of reinforced concrete (RC) structures, ultra-high performance concrete (UHPC) materials with better mechanical properties and durability have emerged. Compared with ordinary concrete, UHPC has a dense microstructure [2] and a very low internal porosity [3,4], which makes its internal moisture content lower than that of ordinary concrete, so it has excellent frost resistance and durability [5,6,7]. This provides the possibility to improve the mechanical properties of reinforced concrete structures in freeze–thaw environments. UHPC is wrapped in the outer layer of the RC column to form a UHPC-RC composite column, which can not only give full play to the excellent frost resistance and high compressive strength (more than 120 MPa) of UHPC, improve the service life and bearing capacity of the structure but also reduce the size of the column and save the building space.

In the past few decades, many scholars have studied the mechanical properties and failure mechanism of ordinary concrete and UHPC under freeze–thaw cycles (F-T). Qiu et al. (2020) [8] found that when the number of F-T increases from 0 to 100, the initial tangent modulus and peak secant modulus gradually decrease. Li et al. (2022) [9] used acoustic emission characteristics to find that with the increase of F-T times, the damage degree inside the concrete was greater, showing increasingly obvious brittleness. Cao et al. (2013) [10] found that the larger the number of F-T, the flatter the compressive stress–strain curve and the larger the peak strain. Dong et al. (2021) [11] found that the freeze–thaw cycle destroyed the interface bonding between the fiber and the matrix, which reduced the strain performance of PE-steel fiber hybrid concrete (FRC). Lu et al. (2021) [12] found that among the components of UHPC, the water-binder ratio had the greatest influence on frost resistance. Li et al. (2020) [13] found that the damage of the chloride salt freeze–thaw cycle to reactive powder concrete (RPC) was greater than that of the freshwater freeze–thaw cycle. Zhang et al. (2020) [14] found that freeze–thaw cycles accelerated the development of cracks in ordinary reinforced concrete (RC) columns, and the number of cracks increased with the increase of F-T times. Yang et al. (2022) [15] found that with the increase in freeze–thaw cycles, the ultimate bearing capacity of large eccentric RC columns decreased significantly, while the ultimate bearing capacity of small eccentric RC columns did not change much. Xu (2016) [16] and Rong (2022) [17] found that the stiffness and compressive strength attenuation rate of RC columns increased with the increase in freeze–thaw cycles.

In addition, much research has been carried out on the use of UHPC to increase the mechanical properties of RC columns at room temperature. Gholampour [18] and Farzad [19] compared the RC columns strengthened with UHPC with the RC columns not strengthened and found that the composite columns showed ductile failure, while the RC columns showed brittle failure. The use of UHPC tubes can improve the bearing capacity, strain, and ductility of RC columns without increasing the interface size. Xie [20] found that the axial compressive bearing capacity of UHPC-RC composite columns gradually increased with the increase in UHPC tube thickness. Li [21] found that the bearing capacity of the composite column of pouring the concrete core first and pouring the UHPC tube later is higher than that of pouring the UHPC tube first and pouring the core concrete later. Zhang [22] and Shan [23] found that increasing the steel fiber content and stirrup ratio of UHPC tubes can effectively improve the peak load and ductility of UHPC-RC composite columns. Zeng found that the failure mode of UHPC-RC composite columns is a brittle failure when using ordinary stirrups [24], while it is a ductile failure when using high-strength stirrups [25].

From the above research, it can be seen that at present, the research on concrete and UHPC under freeze–thaw cycles is only limited to the field of materials, and the research on UHPC-RC composite short columns is mainly concentrated in the field of normal temperature. The axial compression performance of UHPC-RC composite short columns under freeze–thaw cycles is rarely reported. However, under the action of freeze–thaw cycles, it will not only reduce the strength of concrete but also reduce the bond strength between UHPC and concrete and between concrete and steel bars [26,27,28], which may accelerate the deterioration of the axial compression performance of UHPC-RC composite columns. Therefore, it is necessary to explore whether UHPC-RC is applicable under freeze–thaw cycles.

Therefore, to explore the applicability of UHPC-RC composite columns in freeze–thaw environment and the change in axial compression performance, axial compression tests were carried out on UHPC-RC composite columns with high-strength stirrups under different freeze–thaw cycles (0, 100, 200, 300 cycles) and different stirrup spacing (35, 70, 105 mm). The changes of hoop strain on the surface of stirrups and composite columns during loading were analyzed. The effects of freeze–thaw cycles and stirrup spacing on the peak load, ductility coefficient, and initial elastic modulus of composite short columns were analyzed. The peak load prediction formula suitable for different freeze–thaw cycles was established. This study can provide a theoretical and experimental basis for the design and transformation of UHPC-RC composite columns for piers and wharf support columns working in cold regions and promote the application of UHPC-RC composite columns in practical engineering.

## 2. Experimental Program

### 2.1. Specimen Design and Preparation

**Figure 1:**
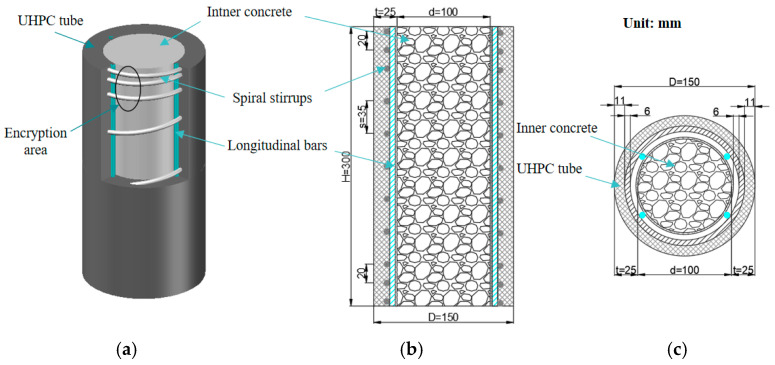
Composite column section. (**a**) Composite column; (**b**) Composite column longitudinal-section; (**c**) Composite column cross-section.

The diameter of the composite column *D* = 150 mm, the height *H* = 300 mm, and the internal concrete diameter *d* = 100 mm. The UHPC-RC composite column was composed of the outer high-strength stirrup-reinforced UHPC tube and the inner core concrete, as shown in Figure 1. To prevent the first damage at both ends of the composite column during the loading process, the stirrups were encrypted within 40 mm at the ends of both sides of the composite column, and the spacing of the encrypted stirrups was 20 mm, as shown in Figure 1a. The composite column stirrup was the 6 mm high-strength 65 Mn spring steel spiral stirrup (Shanghai Shanren Precision Spring Factory, Shanghai, China), the longitudinal reinforcement was the HRB400 steel bar (Shanghai Yixuan Industry, Shanghai, China), and the thickness of the protective layer was 11 mm, as shown in Figure 1c.

**Figure 2:**
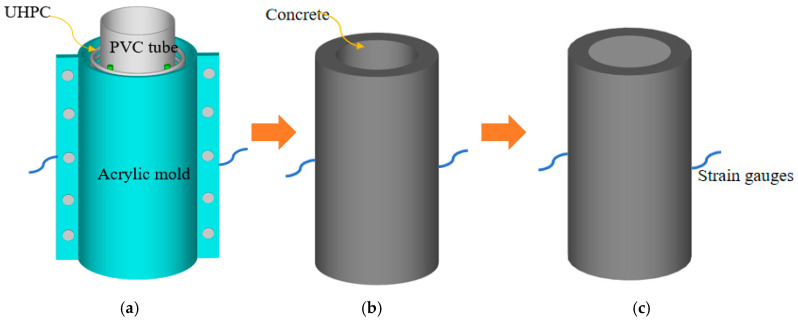
UHPC-RC short column pouring process. (**a**) Mold assembly; (**b**) UHPC tube; (**c**) UHPC-RC short column.

The pouring process of the composite column adopted the method of pouring the UHPC shell first and then pouring the internal concrete. To maintain the integrity of the steel strain gauge, a detachable transparent acrylic plate was used as the outermost mold, and the PVC pipe was used as the internal mold (Figure 2a). The tied steel cage was placed between the internal and external molds, then the UHPC was poured between the internal and external molds, and finally, the internal concrete was poured. After the pouring of the composite column was completed, it was placed in the standard maintenance room for 28 days.

### 2.2. Experimental Design

#### 2.2.1. Freeze–Thaw Cycle Test

**Figure 3:**
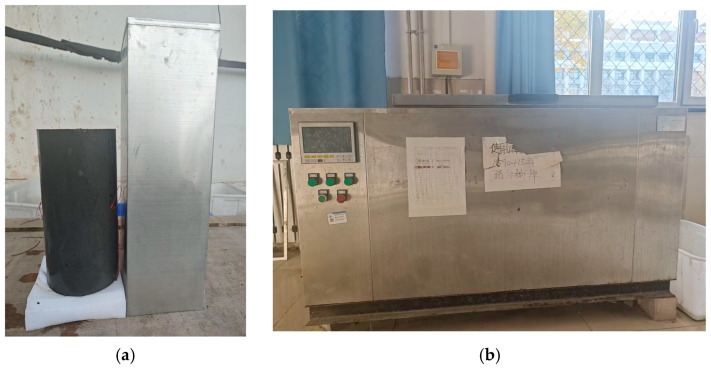
Freeze–thaw test equipment. (**a**) Composite column freeze–thaw mold; (**b**) Freeze-thaw machine.

The model JB-TDRF-28F concrete rapid freeze–thaw cycle test machine, produced by Shanghai Jiaben Test Equipment Co., Ltd. (Shanghai, China), was used. The specimens were tested according to the quick-freezing method in the Chinese standard GB/T50082-2019 [29]. The quick freezing method is carried out by water freezing and water thawing method, and the specific operation is as follows. After the specimen was cured for 24 days, the specimen was taken out from the standard curing room and immersed in fresh water for 4 days. The water surface was 20–30 mm higher than the surface of the specimen during immersion. After the immersion was completed, the composite short-column specimens were placed in an iron bucket (Figure 3a). The water in the iron bucket did not exceed the specimen 5 mm, and then the iron bucket was placed in a freeze–thaw machine (Figure 3b) for freeze–thaw cycles.

The number of freeze–thaw cycles (0, 100, 200, 300 cycles) and the spacing of stirrups (35, 70, 105 mm) were used as variables to study the axial compression performance of UHPC-RC composite columns under freeze–thaw action. For each group of three specimens, test conditions such as Table 1.

#### 2.2.2. Loading and Measuring Setup

**Figure 4:**
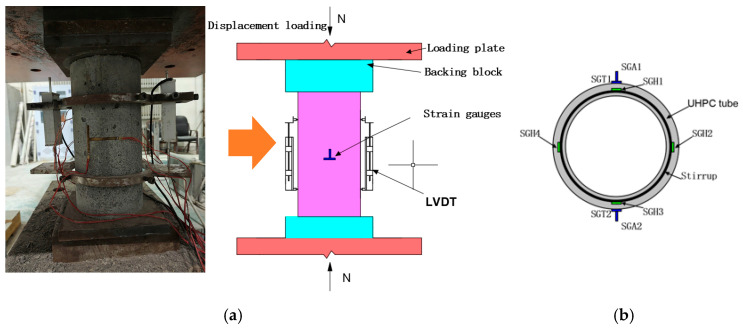
Test setup. (**a**) Test setup; (**b**) Strain gauge arrangement.

The axial compression test was carried out on a 500 T microcomputer-controlled electro-hydraulic servo pressure testing machine (Tianshui Hongshan Testing Machine Co., Ltd., Tianshui, China). According to the requirements of the Chinese standard GB 50010-2010 [30], the loading rate was 0.5 mm/min. Two displacement sensors were used to measure the axial displacement; the acquisition frequency was 1 Hz, and the gauge distance of the axial strain test frame was *h* = 150 mm (Figure 4). Four strain gauges (SGA1-2, SGT1-2) were pasted on the surface of the specimen to measure the vertical and hoop strain. Four steel strain gauges (SGH1-4) were pasted on the center line of stirrups in the middle of the composite column to measure the strain of stirrups during loading.

### 2.3. Test Materials and Properties

**Figure 5:**
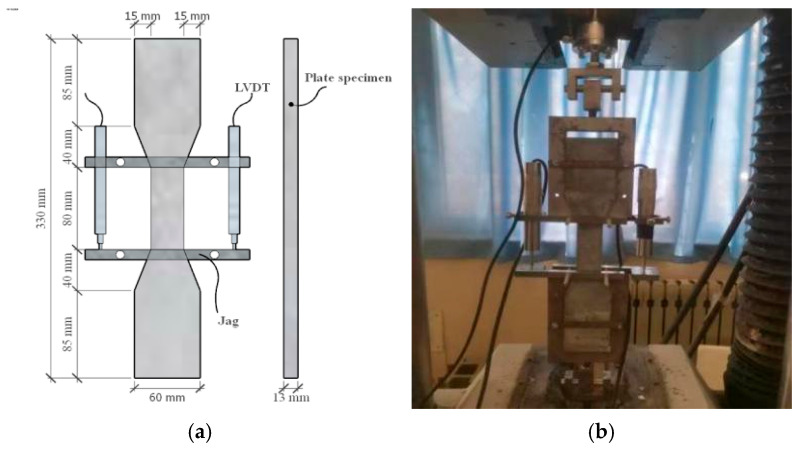
UHPC tensile test. (**a**) Specimen size; (**b**) Loading figure.

The materials for preparing UHPC included P.II 52.5R Portland cement (Anhui Conch Cement Co., Ltd., Wuhu, China), Grade I fly ash (FA, Tianjin Yandong Haotian Mineral Products Co., Ltd., Tianjin, China), silica fume (SF, Gansu Sanyuan Silicon Material Co., Ltd., Lanzhou, China), river sand (particle size of 0~2.36 mm, fineness modulus of 2.46, Dazhi Cement Sand Stone, Tianjin, China), polycarboxylate superplasticizer (Shanghai Kaiyin Chemical Co., Ltd., Shanghai, China), and straight round copper-plated steel fiber (Beckart Applied Materials Technology (Shanghai) Co., Ltd., Shanghai, China). The materials for preparing C40 concrete included P·O 42.5 Portland cement (Hebei Jiegui Mineral Products Co., Ltd., Handan, China), river sand (particle size of 0.63~0.85 mm), stone (particle size 5~20 mm, Dazhi Cement Sand Stone, Tianjin, China), and the mix ratio is shown in Table 2. The compressive strength of UHPC was tested by cylinder (φ100 mm × 200 mm), and cube (100 mm), and the tensile strength was measured by the dog bone specimen (Figure 5a). At the same time, the cube (100 mm) was prepared to measure the compressive strength of the concrete material. According to the European standard [31], the axial compressive strength of the cylinder was 0.79 times the compressive strength of the cube. The material properties under different working conditions are shown in Table 3. The yield strength of high-strength stirrups in the composite column was 1248 MPa, the yield strain was 5500 με, and the yield strength of HRB400 longitudinal reinforcement was 457 MPa.

## 3. Test Results and Discussions

### 3.1. Failure Modes

**Figure 6:**
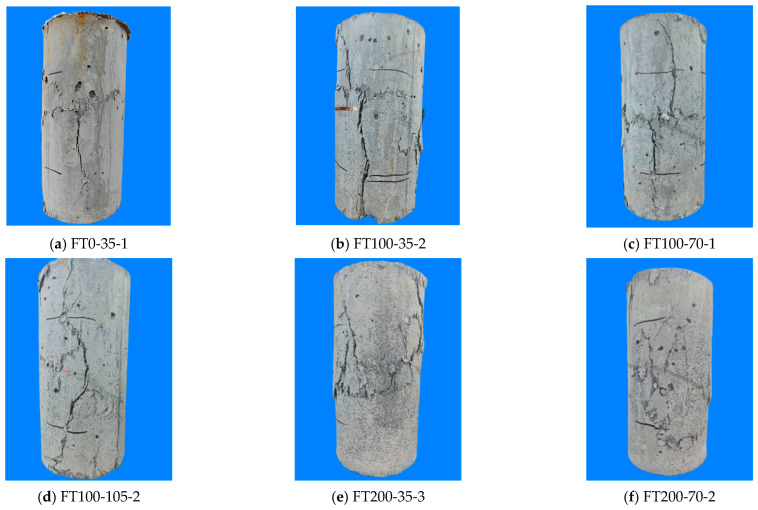

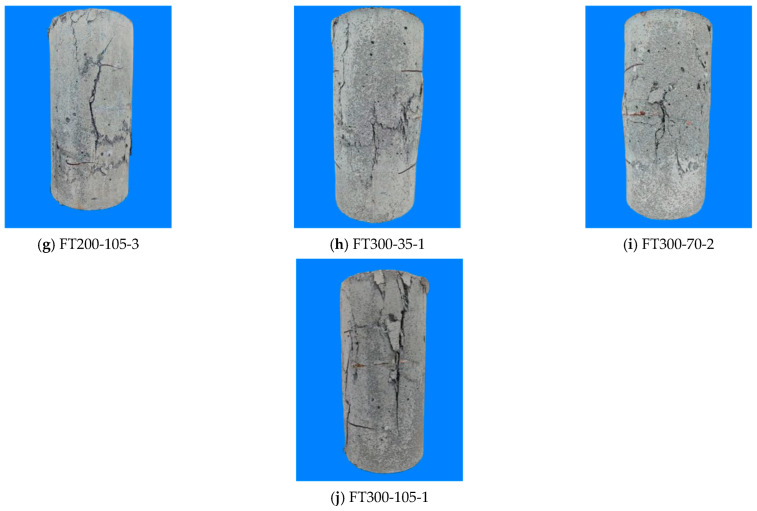
Failure patterns of specimens under different working conditions.

Figure 6 shows the failure modes of all specimens under different freeze–thaw cycles and stirrup spacing. The FT100-70-1 specimen was taken as an example for analysis. At the initial stage of loading, the specimen was in the elastic stage, and there were no obvious cracks on the surface of the specimen. With the increase of load, when the load reached 90% of the peak load, cracks began to appear in the middle and upper parts of the specimen surface. With the continuous increase of load, the obvious tearing sound of steel fiber pull-out can be heard, and high-strength stirrups and UHPC pipes began to constrain the internal concrete. As the load continues to increase, the cracks in the middle of the specimen increase. When the peak load is reached, a penetrating oblique crack is formed from top to bottom. As the load continues to decrease slowly, the cracks continue to expand. After the peak load, the bearing capacity began to decrease, and the outer drum of the middle shell of the specimen was obvious. When the decrease reached 80% of the peak load, the loading was stopped.

From Figure 6, it can be seen that all UHPC-RC composite columns are mainly in shear failure when they are destroyed, and the UHPC shell has not fallen off. Even after 300 freeze–thaw cycles, the UHPC shell still has good bonding performance with the internal concrete and does not fall off. This shows that UHPC can still exert its own high durability and crack resistance of steel fibers even after experiencing freeze–thaw cycles, prevent the falling off of the protective layer, and not expose the steel bars, thereby improving the service life of reinforced concrete columns.

From Figure 6, it can be seen that the number of cracks in FT200-35-3 (Figure 6d) and FT300-35-1 (Figure 6g) specimens is significantly more than that in FT100-35-2 specimens, and the outer drum of the UHPC shell in the middle is more obvious, and the damage of specimens is more serious. This is because, under the action of freeze–thaw cycles, the internal concrete, and UHPC are damaged. As the number of freeze–thaw cycles increase, the damage gradually increases, making the specimen more serious when it is destroyed [14]. By observing the FT200-35-3 (Figure 6d) specimen, it can be found that the number of cracks during failure is significantly higher than that of the FT200-105-3 (Figure 6f) specimen, and the UHPC shell in the middle is more obvious, which is related to the fact that the 35 mm stirrup enhances the restraint force of the composite column.

### 3.2. Axial Load-Strain Curves

**Figure 7:**
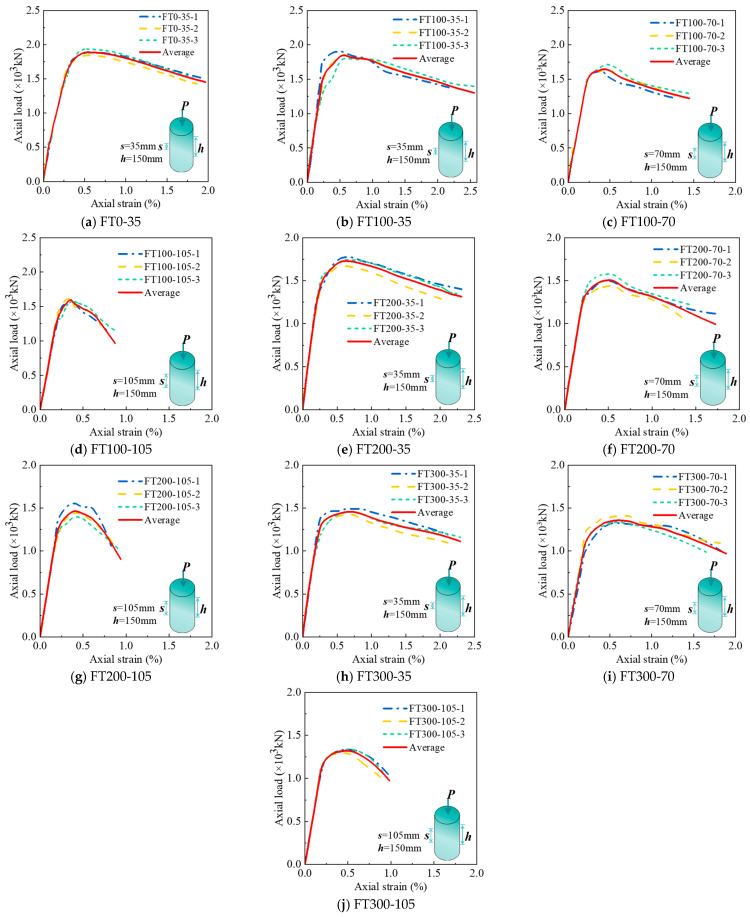
Axial load–strain curves.

Figure 7 draws the axial load-strain curves under different freeze–thaw cycles and stirrup spacing and gives the average curves of the three groups of curves. The peak load $N_{u,t}$ and peak strain $\varepsilon_{cc}$ data obtained by the curve are summarized in Table 4. It can be seen from Figure 7 that the axial load–strain curve shows three stages, which are the I linear stage, II nonlinear stage, and III stable decline stage.

**Figure 8:**
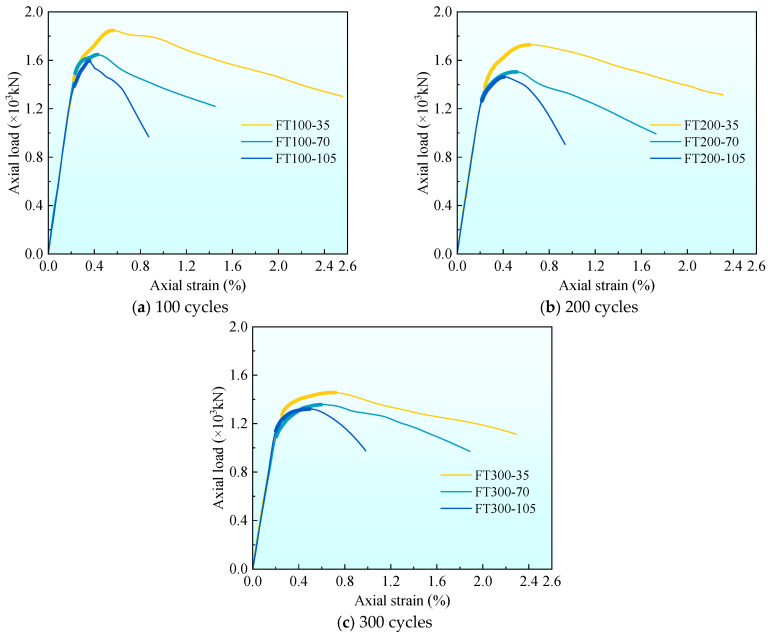
The average axial load–strain curve of each group under the different stirrup spacing.

Figure 8 gives the axial load-strain curves under different stirrup spacing. To facilitate the comparison of the characteristics of each stage of the curve, the average curves of each group are listed in Figure 8 for comparison. It can be seen from Figure 8 that in stage I (linear stage), all curves show an upward state. At the end of stage I, the first crack on the surface of the composite column appears, and the axial load at this time is the cracking load, which accounts for 80–90% of the peak load. There is no obvious rule for the influence of stirrup spacing on cracking load. In the stage II (nonlinear stage), the cracks continue to develop. With the increase of stirrup spacing, the nonlinear stage becomes shorter, and the peak strain decreases gradually. At 100 freeze–thaw cycles, the peak strain $\varepsilon_{cc}$ of 35 and 70 mm stirrup spacing specimens increased by 55.6% and 25.0%, respectively, compared with 105 mm stirrup spacing specimens. At 200 freeze–thaw cycles, the peak strain $\varepsilon_{cc}$ increased by 47.6% and 21.4%, respectively, and at 300 freeze–thaw cycles, the peak strain $\varepsilon_{cc}$ increased by 44.9% and 20.4%, respectively. The peak strain $\varepsilon_{cc}$ increases with the increase of stirrup spacing because the smaller the stirrup spacing, the more uniform the constraint on the internal concrete. In stage III (stable decline stage), it can be found that with the increase of stirrup spacing, each curve becomes shorter and steeper. This is because when the composite column is compressed and deformed, the more the number of stirrups, the better the deformation resistance effect, making the stable decline stage longer and slower.

**Figure 9:**
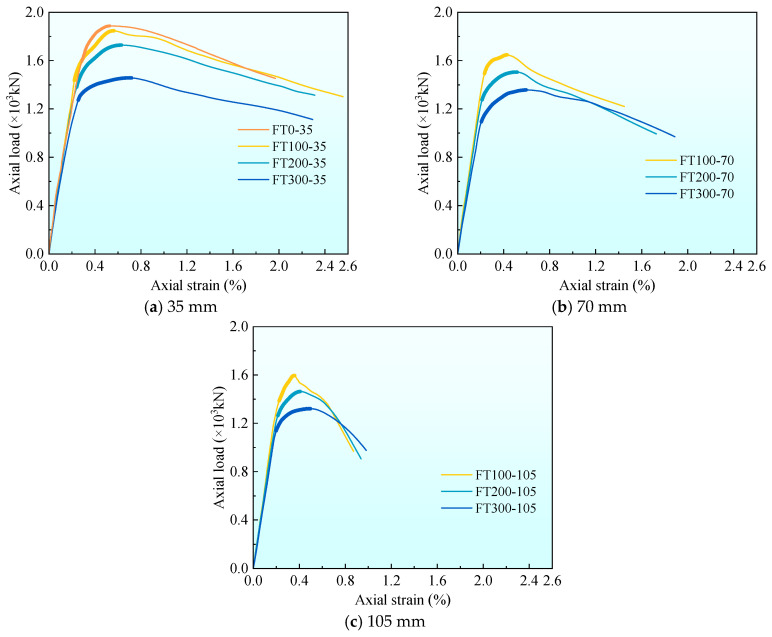
The average axial load–strain curve of each group under different freeze–thaw cycle coefficients.

Figure 9 shows the axial load-strain curves under different freeze–thaw cycles. It can be found from Figure 9 that the slope of the curve is different in the first stage (linear stage). As the number of freeze–thaw cycles increase, the slope of the curve gradually decreases, which is caused by the decrease of the elastic modulus of UHPC and concrete materials under freeze–thaw cycles. At the end of stage I, the cracking load accounts for 80–90% of the peak load. As the number of freeze–thaw cycles increase, the cracking load gradually decreases, which is related to the fact that the freeze–thaw cycle reduces the strength of concrete and UHPC. In stage II (nonlinear stage), it can be found that with the increase in the number of freeze–thaw cycles, the nonlinear stage is longer. This is because, with the increase in the number of freeze–thaw cycles, more micro-cracks are generated inside the concrete and UHPC, resulting in a decrease in peak stress and an increase in peak strain. This phenomenon is more obvious when the stirrup spacing is 105 mm rather than 35 mm. This is because the performance of concrete materials deteriorates under the action of freeze–thaw cycles. The larger the stirrup spacing, the smaller the confinement effect of stirrups on concrete. When the stirrup spacing is 35 mm, the peak strain $\varepsilon_{cc}$ at 200 and 300 freeze–thaw cycles is 10.7% and 26.8% higher than that at 100 freeze–thaw cycles, 13.3% and 28.9% at 70 mm, and 16.7% and 36.1% at 105 mm, respectively. With the increase in the number of freeze–thaw cycles, the peak strain gradually increases, which is related to the development of pores and microcracks in concrete [9] and UHPC promoted by freeze–thaw cycles. In stage III (stable decline stage), the curve becomes longer and slower with the increase of freeze–thaw cycles, especially when the stirrup spacing is 105 mm (Figure 9c).

### 3.3. Axial Load–Stirrup Strain Curves

**Figure 10:**
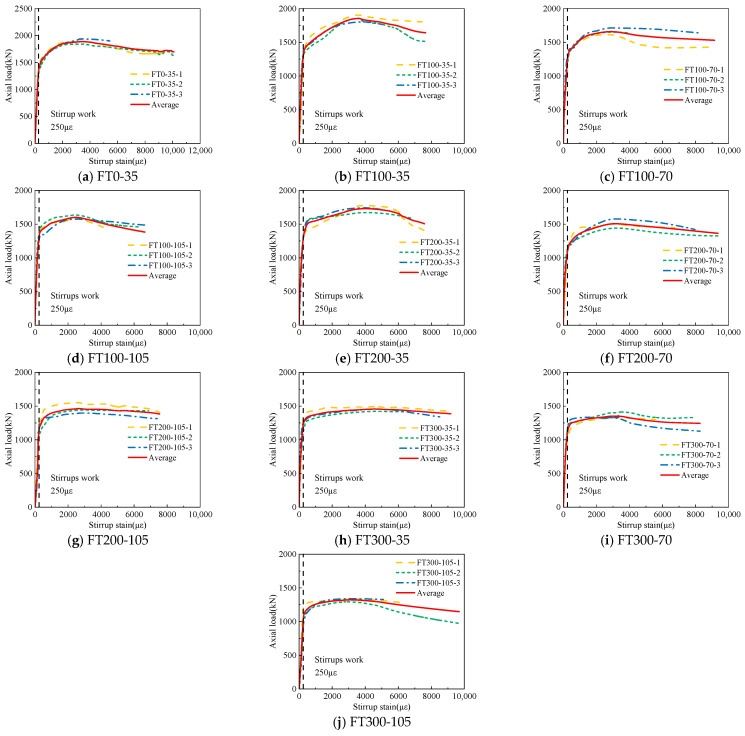
Axial load–stirrup strain curves of freeze–thaw cycles.

Figure 10 gives the axial load–stirrup strain curves of three specimens under each working condition. The stirrup strain in the figure is taken from the mean value of the four strain gauges attached to the surface of the stirrup. The stirrup strain at the peak load is listed in Table 4. Due to the large number of specimens, to facilitate the comparison of the curves, the average curves of each group of specimens given in Figure 10 are compared.

**Figure 11:**
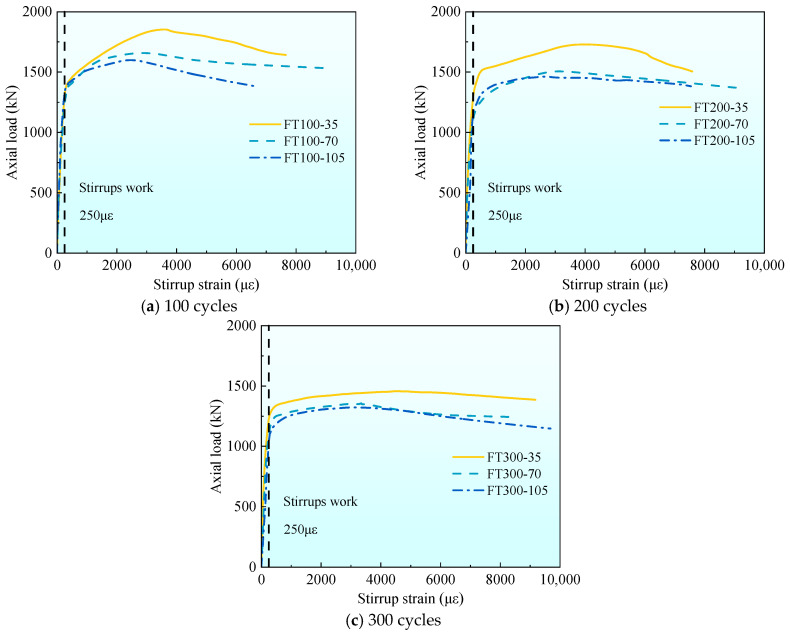
The average axial load–stirrup strain curve of each group under the different stirrup spacing.

Figure 11 gives the average curve comparison figure of axial load-stirrup strain with different stirrup spacing under different freeze–thaw cycles. From Figure 11, it can be seen that the time of action of stirrups in all freeze–thaw cycle specimens is near 250 με, which is similar to the ultimate tensile strain of concrete [32]. This is because the UHPC tubes cracked later on the surface of the combined columns due to the crack resistance of the steel fiber during the loading process, and the UHPC tube has not fallen off, which can provide better restraint for the internal concrete and limit the hoop deformation of the concrete. With the increase of stirrup spacing, the peak load of stirrups increases gradually. For all UHPC-RC composite column specimens, when the stirrups play a role, the stirrup strain increases rapidly until the test stops or the stirrups break.

Combined with Table 4 and Figure 11, it can be seen that when the number of freeze–thaw cycles is the same, with the increase of stirrup spacing, the stirrup strain at the peak value gradually increases. The peak strain of stirrups in 35 mm specimens at 100 freeze–thaw cycles is 23.9% and 44.8% higher than that of 70 mm and 105 mm specimens, respectively. It increased by 27.6% and 46.6%, respectively, at 200 freeze–thaw cycles and increased by 34.2% and 50.5%, respectively, at 300 freeze–thaw cycles. With the increase in the number of freeze–thaw cycles, the influence of stirrup spacing on the peak stirrup strain is greater. This is because the greater the number of freeze–thaw cycles, the greater the internal damage of concrete, the worse the constraint effect of stirrups on internal concrete, and the greater the lateral deformation of the specimen.

**Figure 12:**
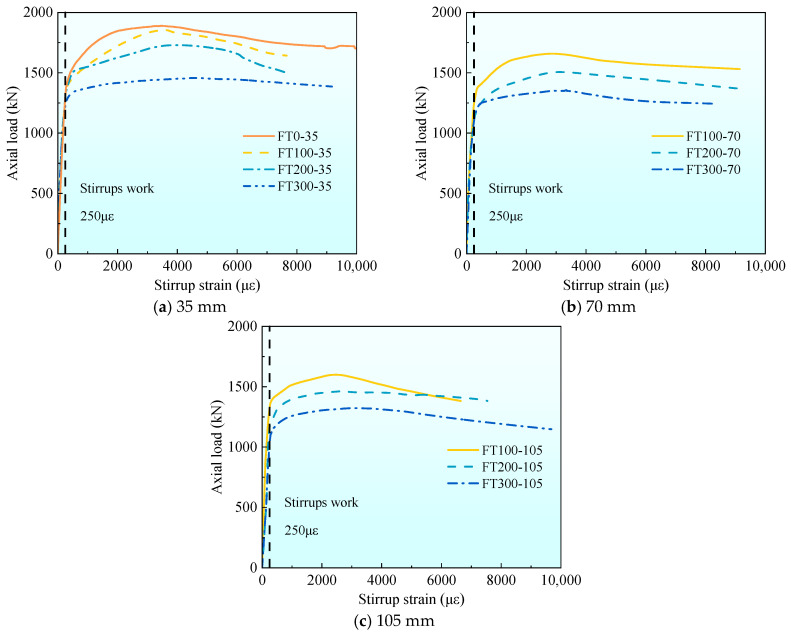
The average axial load–stirrup strain curve of each group under different freeze–thaw cycle coefficients.

Figure 12 shows the average curve comparison figure of axial load-stirrup strain under different freeze–thaw cycles with different stirrup spacing. It can be seen from Figure 12 that under the same stirrup spacing, as the number of freeze–thaw cycles increase, the proportion of the load when the stirrup plays a role in the peak load increases. Combined with Table 4, it can be seen that as the number of freeze–thaw cycles increase, the peak stirrup strain gradually increases. When the stirrup spacing is 35 mm, the peak stirrup strain of the 200 and 300 freeze–thaw cycles is 10.6% and 25.5% higher than that of the 100 specimens, respectively. The 200 freeze–thaw cycles increased by 7.3% and 15.8%, respectively, and the 300 freeze–thaw cycles increased by 9.2% and 20.7%, respectively. This phenomenon occurs because the more the number of freeze–thaw cycles, the greater the degree of damage to the concrete, the greater the axial strain, and the greater the lateral expansion.

### 3.4. Axial Load–Hoop Strain

**Figure 13:**
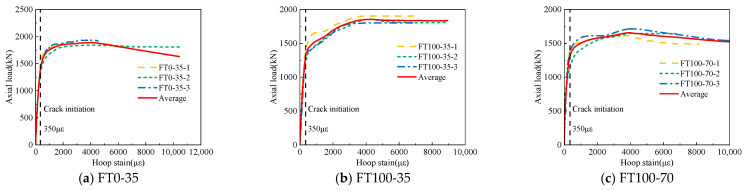

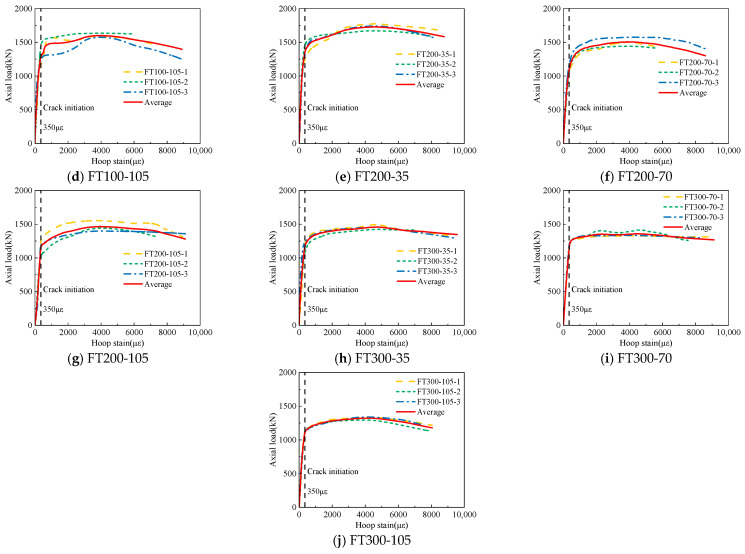
Axial load-hoop strain curves of freeze–thaw cycles.

Figure 13 gives the axial load–hoop strain curves of three specimens under each working condition and gives the average curve of each group of specimens. Table 4 gives the hoop strain value of the surface of the composite short column at the peak load. The hoop strain value is taken from the average value of the two transverse strain gauges on the surface of the composite column.

**Figure 14:**
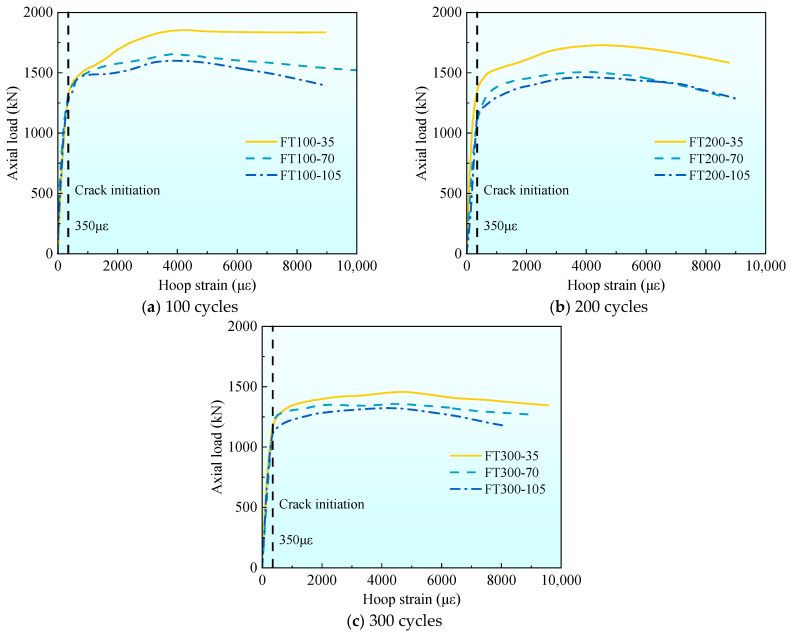
The average axial load–hoop strain curve of each group under the different stirrup spacing.

Figure 14 shows the average curve comparison figure of axial load-hoop strain with different stirrup spacing under different freeze–thaw cycles. It can be seen from Figure 14 that the strain of UHPC specimens increases suddenly at 350 με, which indicates that the composite short column cracks after 350 με, and the cracks develop rapidly after cracking until the crack width is too large to make the strain gauge fail. Combined with the value of hoop strain at peak load given in Table 4, it can be seen that the peak strain of stirrups in 35 mm specimens increased by 6.5% and 11.9%, respectively, compared with 70 mm and 105 mm specimens at 100 freeze–thaw cycles, and increased by 10.2% and 15.5% respectively at 200 freeze–thaw cycles, and increased by 7.6% and 15.6% respectively at 300 freeze–thaw cycles. The smaller the stirrup spacing, the hoop strain increases under each freeze–thaw cycle. The reason for this phenomenon is the same as the reason for the increase of stirrup strain at peak load.

**Figure 15:**
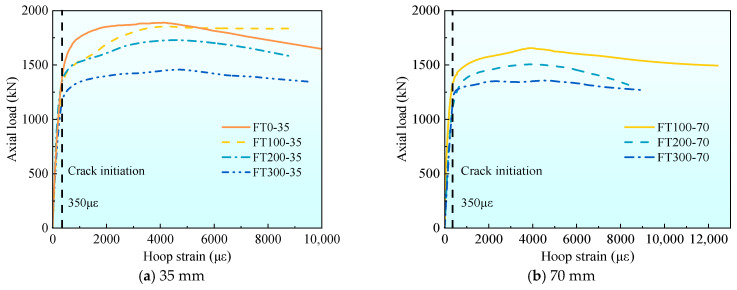

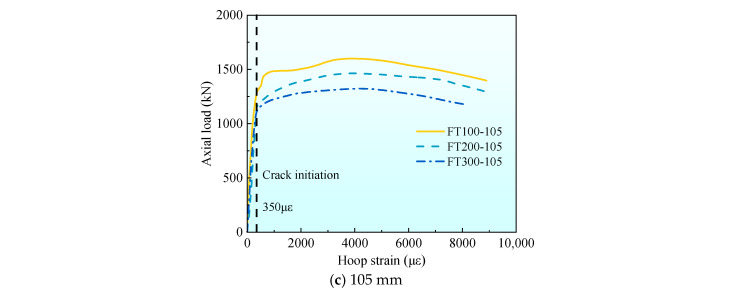
The average axial hoop–stirrup strain curve of each group under different freeze–thaw cycle coefficients.

Figure 15 shows the average curve comparison figure of axial load-stirrup strain under different freeze–thaw cycles with different stirrup spacing. From Figure 15 and the hoop strain at peak load given in Table 4, it can be seen that when the stirrup spacing is 35 mm, the peak strain of stirrups in 200 and 300 freeze–thaw cycles is 6.5% and 10.7% higher than that of 100 specimens, respectively. When the stirrup spacing is 70 mm, it increases by 2.9% and 9.6%, respectively, and when the stirrup spacing is 105 mm, it increases by 3.1% and 7.1%, respectively. When the stirrup spacing is the same, as the number of freeze–thaw cycles increase, the hoop strain at peak load gradually increases.

### 3.5. Peak Load ${\;N}_{u,t}$, Initial Elastic Modulus E, Ductility Coefficient  μ, Toughness Index T.I.

**Figure 16:**
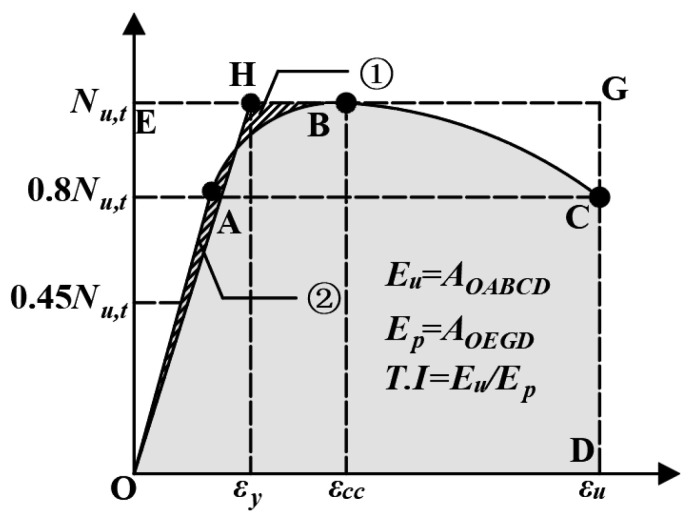
Definition of E, μ, T.I.

Table 4 summarizes the mechanical properties of all UHPC-RC composite short columns. The initial elastic modulus E takes the slope of the curve at 0.45$N_{u,t}$. μ is defined as the ratio of the ultimate strain $\varepsilon_{u}$ to the yield strain $\varepsilon_{y}$, that is, $\mu ={\;\varepsilon }_{u}/\varepsilon_{y}$ [23]. As shown in Figure 16, the yield strain $\varepsilon_{y}$ is determined by an equivalent bilinear response curve equal to the area of the response curve, i.e., ① and ② are equal in area. The strain at point H is the yield strain.$\varepsilon_{u}$ is the strain at 80% of the peak load, i.e., point C. The toughness index T.I. is the ratio of $E_{u}$ to $E_{p}$, as shown in Figure 16 $E_{u}$ and $E_{p}$ are the areas of OABCD and OEGD, respectively [33].

#### 3.5.1. Effects of Different Stirrup Spacing (35, 70, 105 mm)

**Figure 17:**
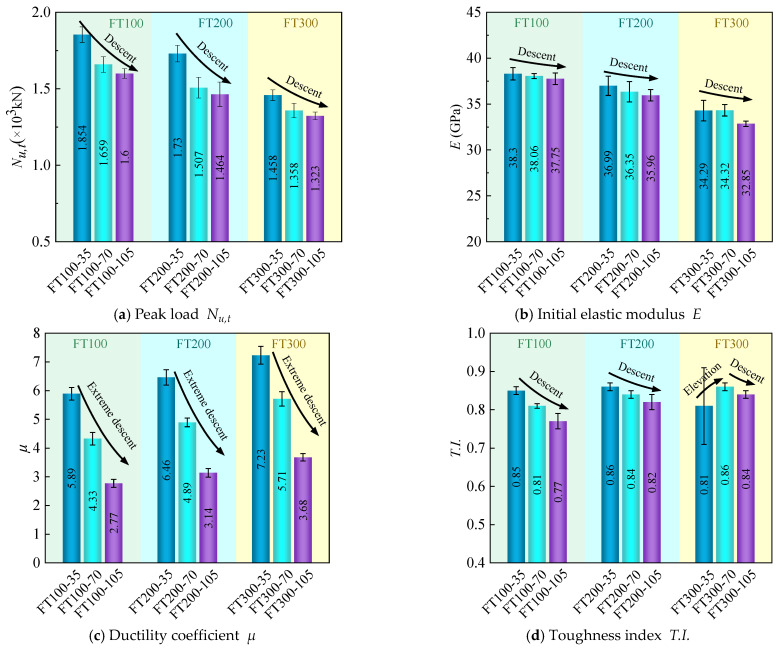
Test results of mechanical properties under different stirrup spacing.

Figure 17 shows the test results of the mechanical properties of composite columns with different stirrup spacing under different freeze–thaw cycles. It can be seen from Figure 17a that the peak load $N_{u,t}$ of specimens with 35 mm and 70 mm stirrup spacing increased by 15.9% and 3.7%, respectively, compared with that of specimens with 105 mm stirrup spacing during 100 freeze–thaw cycles, increased by 18.2% and 2.9%, respectively, during 200 freeze–thaw cycles, and increased by 10.2% and 2.7% respectively during 300 freeze–thaw cycles. Under these three freeze–thaw cycles, the peak load shows that the peak load $N_{u,t}$ gradually increases with the increase of stirrup spacing. This is related to the increase of stirrup spacing, which increases the constraint capacity of stirrups and makes the constraint effect of internal concrete better.

From Figure 17b, it can be seen that the reduction of the initial elastic modulus E of the specimens with 70 and 105 mm stirrup spacing is less than 4.2% under three different freeze–thaw cycles, especially when the number of freeze–thaw cycles is 100, the maximum reduction of the initial elastic modulus E is 1.5%. The stirrup spacing has little effect on the initial elastic modulus because the change of the initial elastic modulus is mainly determined by the elastic modulus of the material, and the change of the stirrup spacing does not change the elastic modulus of the material [22].

It can be seen from Figure 17c that the ductility coefficient μ of specimens with 35 mm and 70 mm stirrup spacing increased by 112.6% and 56.3%, respectively, compared with that of specimens with 105 mm stirrup spacing during 100 freeze–thaw cycles, increased by 105.7% and 55.7% respectively during 200 freeze–thaw cycles, and increased by 96.5% and 55.2% respectively during 300 freeze–thaw cycles. With the increase of stirrup spacing, the ductility coefficient μ increased gradually. It can be found that with the increase of freeze–thaw cycles, the effect of stirrup spacing on the increased rate of ductility coefficient μ is smaller. This is because under the action of freeze–thaw cycles, water in the pores of concrete will alternate between liquid and solid, and the shrinkage and expansion of water molecules will cause alternating shrinkage and expansion of concrete [34], thus reducing the bond strength between steel bars and UHPC, which will weaken the confinement effect of stirrups on internal concrete and reduce the effect of stirrup spacing on ductility coefficient μ.

It can be seen from Figure 17d that the toughness index T.I. shows the same change rule as the ductility index in Figure 17c. During 300 freeze–thaw cycles, with the increase of stirrup spacing, the toughness index T.I. increases first and then decreases, which may be due to the error caused by the FT300-35-3 specimen.

#### 3.5.2. Effect of Different Freeze–Thaw Cycles (0, 100, 200, 300 Cycles)

**Figure 18:**
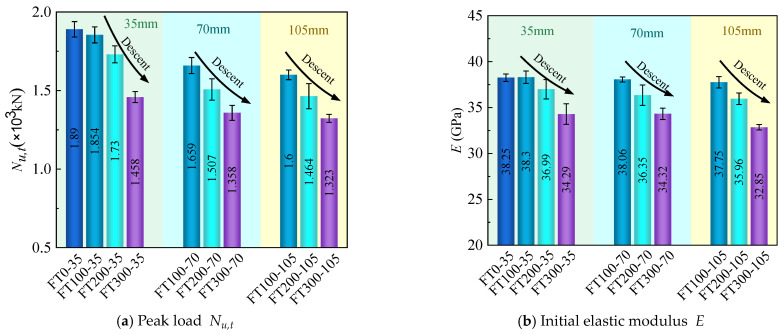

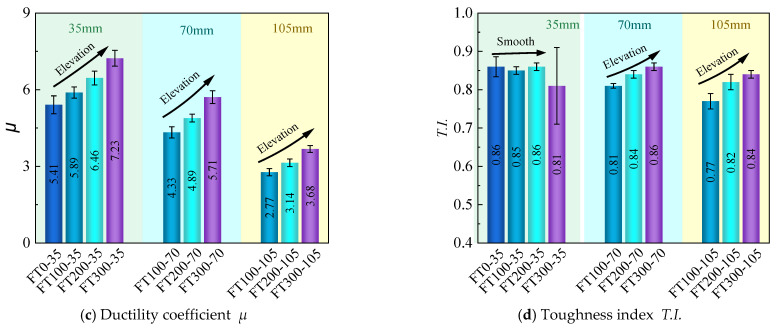
Test results of mechanical properties under different freeze–thaw cycles.

Figure 18 shows the test results of the mechanical properties of composite short columns with different freeze–thaw cycles under different stirrup spacings. It can be seen from Figure 18a that the peak load at 100 freeze–thaw cycles is only 1.9% lower than that at 0 freeze–thaw cycles, and the freeze–thaw cycles before 100 freeze–thaw cycles have little effect on the peak load of composite columns. When the stirrup spacing is 35 mm, compared with the specimens of 100 freeze–thaw cycles, the peak load $N_{u,t}$ of the specimens of 200 and 300 freeze–thaw cycles decreased by 6.7% and 21.4%, respectively, when the stirrup spacing is 70 mm, the peak load $N_{u,t}$ decreased by 9.2% and 18.1%, respectively, when the stirrup spacing is 105 mm, the peak load $N_{u,t}$ decreased by 8.5% and 17.3%, respectively. As the number of freeze–thaw cycles increase, the peak load $N_{u,t}$ of the composite short column gradually decreases. This is because there are certain microcracks and pores in concrete and UHPC. Under the action of freeze–thaw cycles, the water in the pores will convert between liquid and solid, and the resulting expansion force [35] will accelerate the development of microcracks. At the same time, in the process of water freezing, the water in the macropores will freeze first so that the unfrozen water will migrate to the frozen area to form osmotic pressure [36], and the generated osmotic pressure will also accelerate the development of microcracks, thus accelerating the destruction of concrete and UHPC materials. At the same time, for UHPC-RC composite columns, due to the combination of UHPC and concrete materials, freeze–thaw cycles will reduce the bond strength between UHPC and concrete interface [26,27], thereby reducing the peak load of composite columns.

From Figure 18b, it can be seen that the initial elastic modulus of the composite column is little affected before 100 freeze–thaw cycles. When the stirrup spacing is 35 mm, compared with the specimen of 100 freeze–thaw cycles, the initial elastic modulus E of the specimen of 200 and 300 freeze–thaw cycles is reduced by 3.4% and 10.5% respectively; when the stirrup spacing is 70 mm, it is reduced by 4.5% and 9.8% respectively, and when the stirrup spacing is 105 mm, it is reduced by 4.7% and 13% respectively. As the number of freeze–thaw cycles increase, the initial elastic modulus E of the composite short column gradually decreases. The increase in the number of freeze–thaw cycles reduce the compressive elastic modulus of UHPC and concrete materials, which reduces the initial elastic modulus E of UHPC-RC composite columns.

It can be seen from Figure 18c that the ductility coefficient of 100 freeze–thaw cycles is 8.9% higher than that of 0 freeze–thaw cycles. When the stirrup spacing is 35 mm, compared with the specimens of 100 freeze–thaw cycles, the ductility coefficient μ of the specimens of 200 and 300 freeze–thaw cycles increase by 9.7% and 22.8%, respectively. When the stirrup spacing is 70 mm, it increases by 12.9% and 31.9%, respectively. When the stirrup spacing is 105 mm, it increases by 13.4% and 32.9%, respectively. The ductility coefficient increases with the increase of freeze–thaw cycles. The toughness index T.I. (Figure 18d) also increases with the increase in the number of freeze–thaw cycles. The reason for the increase of ductility coefficient μ and toughness index T.I. is mainly due to the increase of porosity and micro-cracks in concrete and UHPC materials after freeze–thaw cycles, which reduces the stiffness of the material. Considering the influence of the number of freeze–thaw cycles on the peak load $N_{u,t}$ and ductility coefficient μ, it can be found that the peak load $N_{u,t}$ of the composite short column decreases, and the ductility coefficient μ increases under the action of freeze–thaw cycles. Therefore, to solve the problem that the peak load of the composite short column decreases under the action of freeze–thaw cycles, the concrete with higher strength can be combined with UHPC to improve the frost resistance of the composite column.

### 3.6. Theoretical Model of Axial Compressive Bearing Capacity of UHPC-RC Composite Columns

#### 3.6.1. Confined Stress

Since the UHPC-RC composite column is a new type of structure, there is no relatively perfect constraint theory to explain it in the freeze–thaw cycle environment. Therefore, to improve the constraint theory of composite columns, this paper is based on Mander [37]. Based on the constraint theory of ordinary reinforced concrete and considering the contribution of UHPC tensile properties to the total constraint stress, the prediction formula of axial compression bearing capacity suitable for freeze–thaw cycle environment is established.

**Figure 19:**
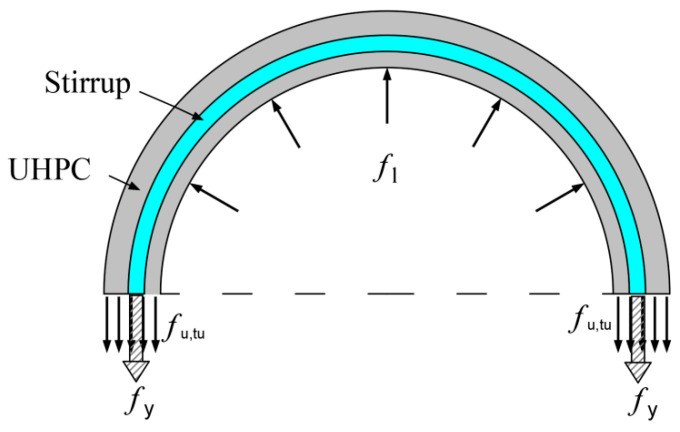
Confined stress of UHPC-RC.

Figure 19 is the constraint stress figure of the internal concrete. From the figure, it can be seen that UHPC can also constrain the internal concrete. According to the equilibrium conditions, it can be obtained:

(1) $$f_{l}^{N}=f_{l,u}^{N}+f_{l,s}=\frac{2f_{u,tu}^{N}t_{u}s}{d_{cor}s}+\frac{2f_{y}A_{sp}}{d_{cor}s}$$

where $f_{l}^{N}$ is the total constraint stress under *N* freeze–thaw cycles; $f_{l,u}^{N}$ is the constraint stress of the UHPC tube under N freeze–thaw cycles; $f_{l,s}$ is the constraint stress provided by high-strength stirrups; $f_{u,tu}^{\;N}$ is the ultimate tensile strength of UHPC under *N* freeze–thaw cycles; $f_{y}$ is the yield stress of stirrups; $d_{cor}$ is the diameter of the core area of the stirrup center line; *s* is stirrup spacing; $t_{u}$ is the thickness of the UHPC tube; $A_{sp}$ is the cross-sectional area of stirrups.

Formula (1) is simplified by introducing the volume stirrup ratio $\rho_{s}$, and the ratio of UHPC tube thickness to ordinary concrete diameter $\rho_{s}$. The calculation formula is as follows:

(2) $$\rho_{s}=\frac{4A_{sp}}{d_{cor}s}$$

(3) $$\rho_{u}=\frac{2t_{u}}{d_{cor}}$$

The mechanical properties of UHPC change after experiencing freeze–thaw cycles. Therefore, for the convenience of calculation, the variation coefficients of UHPC axial compressive strength and ultimate tensile strength $k_{u,co}$, $k_{u,tu}$ are introduced. The calculation formula is as follows:

(4) $$k_{u,co}=\frac{f_{u,co}^{N}}{f_{u,co}}=1-6.04\times 10^{-5}\;N,\;R^{2}=0.98$$

(5) $$k_{u,tu}=\frac{f_{u,tu}^{N}}{f_{u,tu}}=1-2.43\times 10^{-4}\;N,\;R^{2}=0.98$$

Substituting Formulas (2)–(5) into Formula (1), we can obtain the following:

(6) $$f_{l}^{N}=k_{u,tu}f_{u,tu}\rho_{u}+\frac{1}{2}f_{y}\rho_{s}$$

Mander [37] considered that the constraint stress provided by stirrups is not uniform, so he proposed the concept of an effective constraint coefficient $k_{e}$:

(7) $$k_{e}=\frac{A_{e}}{A_{cc}}=\frac{1-s^{\prime }/2d_{cor}}{1-\rho_{cc}}$$

where $s^{\prime }$ is the outer distance of adjacent stirrups; $\rho_{cc}$ is the longitudinal reinforcement ratio of concrete in the core area.

It can be seen from Table 4 that the stirrup strain of all composite column specimens at peak load is less than 5500 με, while the yield strain of high strength stirrup is 5500 με. High-strength stirrups do not yield when the composite column reaches the peak load. If the yield stress of high-strength stirrups is used for calculation, the calculation results will be larger. Therefore, the true stress of stirrups $f_{s}$ should be used to calculate. The calculation formula of $f_{s}$ [38] is as follows:

(8) $$f_{s}=0.01193\frac{k_{e}E_{s}\rho_{s}}{\sigma_{co}}f_{y}+263.9$$

Therefore, the effective constraint stress $f_{l}^{N^{\prime }}$ under *N* freeze–thaw cycles is as follows:

(9) $$f_{l}^{N^{\prime }}=k_{u,tu}f_{u,tu}\rho_{u}+\frac{1}{2}k_{e}f_{s}\rho_{s}$$

#### 3.6.2. Calculation Formula of Axial Bearing Capacity

The axial bearing capacity of the UHPC-RC composite column $N_{u,c}^{T}$ is divided into internal concrete bearing capacity $N_{cc}^{T}$, UHPC shell effective constraint area bearing capacity $N_{u,cc}^{N}$, UHPC shell non-effective constraint area bearing capacity $N_{u,n}^{N}$, longitudinal steel bearing capacity $N_{yv}$ four parts to calculate.

When calculating the bearing capacity $N_{cc}^{T}$ of the internal concrete, the effective constraint stress $f_{l}^{N^{\prime }}$ is the effective constraint stress of the section at the center line of the stirrup. Therefore, to calculate the stress of the internal concrete, the effective constraint stress at the internal concrete must be determined first, so the conversion coefficient $k_{t}$ is introduced.

(10) $$k_{t}=\frac{d_{cor}}{d}$$

The variation coefficient of concrete compressive strength with freeze–thaw cycle $k_{cu}$ is determined by the literature [39], which is the closest to the data in this paper. The specific expression is as follows (11):

(11) $$k_{cu}=\frac{f_{cu}^{N}}{f_{cu}}=1-200f_{cu}^{-3.0355}N$$

where *N* is the number of freeze–thaw cycles; $f_{u,tu}$, $f_{u,tu}^{\;N}$ are the ultimate tensile strength of UHPC without freeze–thaw and after freeze–thaw *N* times; $f_{cu}$, $f_{cu}^{\;N}$ are the compressive strength of concrete cubes without freeze–thaw and after *N* freeze–thaw cycles; the axial compressive strength of concrete $f_{co}$ is 0.79 $f_{cu}$.

The peak stress of internal concrete $f_{cc}^{\;N}$ [38] is expressed as follows:

(12) $$\frac{f_{cc}^{N}}{f_{co}^{N}}=1.21+\sqrt{-0.20+15.16\frac{k_{t}f_{le}^{N^{\prime }}}{f_{co}^{N}}}+2\frac{k_{t}f_{le}^{N^{\prime }}}{f_{co}^{N}}$$

The peak stress $f_{u,cc}^{\;N}$ [40] in the effective constraint area of the UHPC shell is calculated by Formula (13).

(13) $$\frac{f_{u,cc}^{N}}{f_{u,co}^{N}}=-0.812+1.649\sqrt{1+10.579\frac{f_{l,s}^{\prime }}{f_{u,co}^{N}}}+2\frac{f_{l,s}^{\prime }}{f_{u,co}^{N}}$$

Since the UHPC shell may have a tensile and compressive stress state during the loading process, the loss of compressive strength should be considered when calculating the peak bearing capacity of the non-effective restraint zone of the UHPC shell, and the strength attenuation coefficient α should be introduced.

Therefore, the peak bearing capacity of UHPC-RC composite columns $N_{u,c}^{N}$ is calculated as follows:

(14) $$\begin{array}{cc} N_{u,c}^{N} & =N_{cc}^{N}+N_{u,cc}^{N}+N_{u,n}^{N}+N_{yv}\\ & =f_{cc}^{N}A_{cc}+f_{u,cc}^{N}A_{u,cc}+\alpha f_{u,co}^{N}A_{u,n}+f_{y}A_{yv} \end{array}$$

According to the test results of this study, the strength attenuation coefficient α was fitted by the least square method, and α = 0.79 was obtained. The calculation results of the peak bearing capacity of composite columns are shown in Table 5.

**Figure 20:**
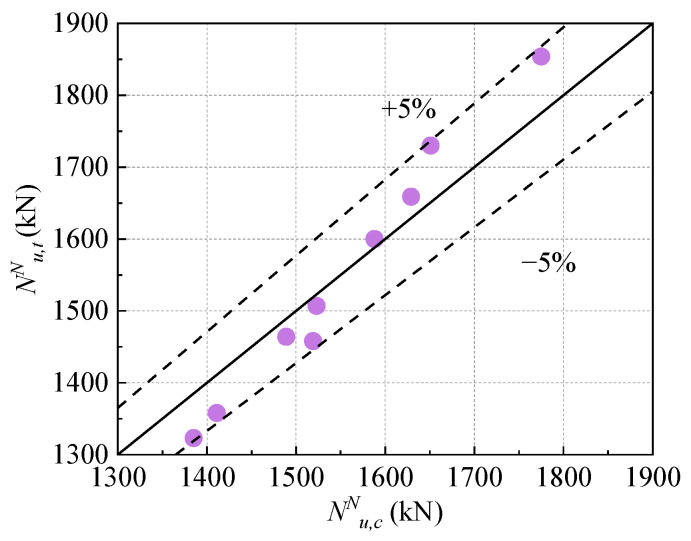
UHPC-RC composite short column peak load calculated value $N_{u,c}^{N}$/test value $N_{u,t}^{N}$ under freeze–thaw cycle.

Figure 20 shows the ratio of the calculated value $N_{u,c}^{N}$ to the experimental value $N_{u,t}^{N}$ under freeze–thaw cycles. It can be seen from the figure that the ratio error between the calculated value $N_{u,c}^{N}$ and the experimental value $N_{u,t}^{N}$ is within 5%. The bearing capacity formula of the UHPC-RC composite column under the freeze–thaw cycle can predict its bearing capacity well.

## 4. Conclusions

The axial compression performance of UHPC-RC composite columns under freeze–thaw cycles was studied by axial compression test. The prediction formula of axial compression bearing capacity under freeze–thaw cycles was established. The main conclusions are as follows:

(1) Even after 300 freeze–thaw cycles, UHPC still has a high bonding force with concrete, ensuring that UHPC does not fall off when the composite column is damaged;
(2) When the stirrup spacing is 35 mm, the peak stirrup strain in 200 and 300 freeze–thaw cycles increase by 10.6% and 25.5%, respectively, compared with that in 100 freeze–thaw cycles, while the peak hoop strain increases by 6.5% and 10.7% respectively. With the increase of freeze–thaw cycles, the increase rate of hoop strain is smaller than that of stirrup strain;
(3) Under the same number of freeze–thaw cycles, with the decrease in stirrup spacing, the peak load $N_{u,t}$ showed a significant downward trend, and the ductility coefficient μ showed an increasing trend. At 300 freeze–thaw cycles, the peak load $N_{u,t}$ of 35 and 75 mm stirrup spacing specimens increased by 10.2% and 2.7%, respectively, compared with 105 mm stirrup spacing specimens, and the ductility coefficient μ increased by 96.5% and 55.2%, respectively;
(4) The effect of freeze–thaw cycles reduce the peak load $N_{u,t}$ and initial elastic modulus E of the composite column, but increases the ductility coefficient μ. When the stirrup spacing is 35 mm, compared with 100 freeze–thaw cycles, the peak load $N_{u,t}$ of 300 freeze–thaw cycles is reduced by 21.4%, the initial elastic modulus E is reduced by 10.5%, and the ductility coefficient μ is 22.8%. It is suggested that the composite columns subjected to long-term freeze–thaw cycles can solve the problem of peak load $N_{u,t}$ and initial elastic modulus E by increasing the strength of the concrete and the thickness of the UHPC shell to improve the frost resistance of composite columns;
(5) By introducing the coefficient of variation of compressive strength of UHPC and concrete under freeze–thaw cycles, and considering the influence of tensile properties of UHPC, the prediction formula of bearing capacity of UHPC-RC composite columns is established. The prediction error of composite columns subjected to 0–300 freeze–thaw cycles is within 5%.

## Figures and Tables

**Table 1:** Freeze–thaw test conditions.

Number	Cycle Time	Stirrup Spacing (mm)	Volume Stirrup Ratio (%)	Specimen Number
FT0-35	0	35	2.649	3
FT100-35	100	35	2.649	3
FT200-35	200	35	2.649	3
FT300-35	300	35	2.649	3
FT100-70	200	70	1.324	3
FT200-70	200	70	1.324	3
FT300-70	300	70	1.324	3
FT100-105	300	105	0.883	3
FT200-105	300	105	0.883	3
FT300-105	300	105	0.883	3

**Table 2:** Mix proportion of concrete and UHPC.

	Water-Binder Ratio	Cement	FA	SF	River Sand	Water	Grave	Steel Fiber	SP
UHPC	0.19	0.55	0.35	0.1	1.2	0.19		2%	0.79%
C40	0.41	1			1.83	0.41	2.29		0.3%

Note: The mix ratio adopts the weight ratio, which is the percentage of the weight of the cementitious material, and the steel fiber is the volume content.

**Table 3:** UHPC and concrete material properties.

Number	Cycle Times	$f_{u,cu}^{\;N}$ (MPa)	$f_{u,co}^{\;N}$ (MPa)	$f_{cu}^{\;N}$ (MPa)	$f_{u,tu}^{\;N}$ (MPa)
FT0-1	0	137.5	117.6	50.8	7.78
FT0-2	0	138.5	119.4	47.3	9.50
FT0-3	0	126.3	110.9	48.6	9.56
Average		134.1	116.0	48.9	8.95
Cov		0.05	0.04	0.04	0.11
FT100-1	100	127.8	118.6	42.2	9.46
FT100-2	100	133.4	116.1	44.5	8.32
FT100-3	100	136.1	110.6	41.6	8.67
Average		132.4	115.1	42.8	8.81
Cov		0.03	0.04	0.04	0.07
FT-200-1	200	131.5	115.2	37.8	8.59
FT200-2	200	126.4	111.7	35.9	9.06
FT200-3	200	136.5	116.4	34.1	7.95
Average		131.5	114.4	35.9	8.53
Cov		0.04	0.02	0.05	0.07
FT300-1	300	125.4	111.6	28.1	8.62
FT300-2	300	134.8	117.3	29.8	7.45
FT300-3	300	132.4	112.9	26.4	8.90
Average		130.9	113.9	28.1	8.32
Cov		0.04	0.03	0.06	0.09

Note: $f_{u,cu}^{\;N}$ is the compressive strength of the UHPC cube under *N* freeze–thaw cycles; $f_{u,co}^{\;N}$ is the axial compressive strength of the UHPC cylinder under *N* freeze–thaw cycles; $f_{cu}^{\;N}$ is the compressive strength of a concrete cube under *N* freeze–thaw cycles; $f_{u,tu}^{\;N}$ is the ultimate tensile strength of UHPC at N freeze–thaw cycles.

**Table 4:** Axial compression test performance summary of freeze–thaw cycles.

Number	$N_{u,t}$ (kN)	$f_{cc}^{\prime }$ (MPa)	$\varepsilon_{cc}$ (%)	μ	E (GPa)	T.I.	$\varepsilon_{s}$ (με)	$\varepsilon_{h}$ (με)
FT0-35-1	1888	106.9	0.55	5.54	38.67	0.89	3543	4137
FT0-35-2	1842	104.2	0.54	5.68	38.22	0.85	3456	4123
FT0-35-3	1939	109.7	0.50	5.02	37.85	0.84	3429	4103
Average	1890	106.9	0.53	5.41	38.25	0.86	3476	4121
FT100-35-1	1904	107.7	0.51	5.66	38.94	0.84	3536	4156
FT100-35-2	1856	105.0	0.57	5.91	38.35	0.85	3623	4245
FT100-35-3	1802	102.0	0.59	6.09	37.61	0.86	3758	4308
Average	1854	104.9	0.56	5.89	38.30	0.85	3639	4236
FT200-35-1	1776	100.5	0.65	6.58	37.25	0.85	4079	4602
FT200-35-2	1671	94.6	0.59	6.15	35.83	0.86	3985	4416
FT200-35-3	1742	98.6	0.62	6.65	37.89	0.87	4006	4513
Average	1730	97.9	0.62	6.46	36.99	0.86	4023	4510
FT300-35-1	1493	84.5	0.68	7.55	33.06	0.88	4495	4578
FT300-35-2	1424	80.6	0.73	6.94	35.25	0.86	4621	4752
FT300-35-3	1457	82.4	0.72	7.19	34.56	0.69	4580	4735
Average	1458	82.5	0.71	7.23	34.29	0.81	4565	4688
FT100-70-1	1612	91.2	0.41	4.34	38.27	0.81	2848	3852
FT100-70-2	1651	93.5	0.48	4.54	38.15	0.82	3026	4076
FT100-70-3	1713	96.9	0.45	4.11	37.75	0.81	2939	4007
Average	1659	93.9	0.45	4.33	38.06	0.81	2938	3978
FT200-70-1	1502	85.0	0.49	5.06	36.64	0.84	3079	4034
FT200-70-2	1441	81.6	0.53	4.82	37.29	0.84	3198	4064
FT200-70-3	1577	89.2	0.52	4.78	35.13	0.83	3181	4185
Average	1507	85.3	0.51	4.89	36.35	0.84	3153	4094
FT300-70-1	1327	75.1	0.56	5.54	34.55	0.87	3343	4282
FT300-70-2	1413	79.9	0.63	5.99	33.62	0.86	3522	4469
FT300-70-3	1334	75.5	0.58	5.59	34.79	0.86	3345	4322
Average	1358	76.8	0.59	5.71	34.32	0.86	3403	4358
FT100-105-1	1589	89.9	0.34	2.66	37.36	0.76	2420	3684
FT100-105-2	1635	92.5	0.36	2.72	38.48	0.76	2513	3832
FT100-105-3	1575	89.1	0.38	2.92	37.42	0.79	2605	3842
Average	1600	90.5	0.36	2.77	37.75	0.77	2513	3786
FT200-105-1	1553	87.9	0.40	2.99	35.53	0.84	2652	3801
FT200-105-2	1443	81.6	0.43	3.15	36.68	0.81	2754	3966
FT200-105-3	1397	79.1	0.42	3.28	35.66	0.81	2826	3945
Average	1464	82.9	0.42	3.14	35.96	0.82	2744	3904
FT300-105-1	1336	75.6	0.51	3.79	33.02	0.84	3085	4130
FT300-105-2	1294	73.2	0.46	3.54	32.52	0.85	2959	3925
FT300-105-3	1339	75.8	0.50	3.70	33.01	0.83	3056	4113
Average	1323	74.9	0.49	3.68	32.85	0.84	3033	4056

Note: $N_{u,t}$ is peak load; $f_{cc}^{\prime }$ is peak stress; $\varepsilon_{cc}$ is peak strain; $\varepsilon_{s}$ is the peak stirrup strain; $\varepsilon_{h}$ is the peak hoop strain; μ is the ductility coefficient; E is the initial elastic modulus; T.I. is the toughness index.

**Table 5:** Summary of bearing capacity of each group of specimens.

Number	$f_{l,u}^{\;N}$ /MPa	$f_{l,s}$ /MPa	$N_{cc}^{\;N}$ /kN	$N_{u,cc}^{\;N}$ /kN	$N_{u,n}^{\;N}$ /kN	$N_{\mathrm{yv}}$ /kN	$N_{u,c}^{\;N}$ /kN	$N_{u,t}^{\;N}$ /kN	$N_{u,c}^{\;N}/N_{u,t}^{\;N}$
FT0-35	3.67	11.28	870	184	788	52	1894	1890	1.00
FT100-35	3.58	11.33	757	183	783	52	1775	1854	0.96
FT200-35	3.49	11.37	638	183	778	52	1651	1730	0.95
FT300-35	3.40	11.42	512	182	773	52	1519	1458	1.04
FT100-70	3.58	5.03	683	-	894	52	1629	1659	0.98
FT200-70	3.49	5.05	582	-	889	52	1523	1507	1.01
FT300-70	3.40	5.07	476	-	883	52	1411	1358	1.04
FT100-105	3.58	2.93	642	-	894	52	1588	1600	0.99
FT200-105	3.49	2.94	548	-	889	52	1489	1464	1.02
FT300-105	3.40	2.95	450	-	883	52	1385	1323	1.05

## Data Availability

The data used to support the findings of this study are available from the corresponding author upon request.
